# How to Promote Wellbeing in Youth Sport: Recommendations From the 2024 Copenhagen Consensus Conference

**DOI:** 10.1111/sms.70121

**Published:** 2025-08-12

**Authors:** Glen Nielsen, Peter Krustrup, Niels Nygaard Rossing, Peter Juul Elsborg, Jesper Nøddesbo, Annemette Krabbe, Imran Rashid, Bodil Borg Høj, Janne Mortensen, Søren Østergaard, Thomas Skovgaard, Knud Aagaard Ryom, Kenneth Aggerholm, Sine Agergaard

**Affiliations:** ^1^ Department of Nutrition Exercise and Sports University of Copenhagen Copenhagen Denmark; ^2^ Department of Sports Science and Clinical Biomechanics Faculty of Health Sciences University of Southern Denmark Denmark; ^3^ Department of Health Science & Technology Aalborg University Aalborg Denmark; ^4^ Center for Clinical Research and Prevention Copenhagen University Hospital—Bispebjerg and Frederiksberg Frederiksberg Denmark; ^5^ Jesper Noeddesbo Coaching and Consulting Denmark; ^6^ Red Barnet, Save the Children Denmark; ^7^ Centre for Digital Health Copenhagen Denmark; ^8^ VIA University College Aarhus Denmark; ^9^ Mental Motion ApS Denmark; ^10^ Centre for Youth Studies Frederiksberg Denmark; ^11^ Department of Public Health, Applied Public Health Research Aarhus University Aarhus Denmark; ^12^ Department of Teacher Education and Outdoor Studies Norwegian School of Sport Sciences Oslo Norway; ^13^ Department of Health Science and Technology Aalborg University Aalborg Denmark

**Keywords:** club sports, leisure, mental health, physical activity, thriving, wellbeing, young people

## Abstract

On August 29 and 30, 2024, eight researchers and six expert practitioners within wellbeing and youth sport were gathered in Copenhagen to reach evidence‐based consensus on how to promote wellbeing in and through youth sports. The consensus process integrated international research‐based knowledge with practice‐based knowledge into statements about key factors influencing wellbeing in youth sport. Based on this knowledge, practice and policy directed statements on how wellbeing in and through sport can be promoted were formulated. The consensus process and the resulting statements were based on a system approach to wellbeing in youth sport and how it can be promoted. Four system levels (the individual, interpersonal, community, and policy levels) were addressed in answering the following questions: (1) What characterizes wellbeing in sport for children and adolescents? (2) What characterizes sport environments that promote children and adolescents’ wellbeing? (3) Which factors are important to consider in efforts to develop wellbeing promoting youth sport environments? (4) How can wellbeing in youth sport be promoted? In total, 19 consensus statement sentences were formulated on how young sports participants (3), parents/caretakers (5), coaches and leaders/managers (5), local sport communities (3) and sports federations (3) can promote wellbeing in youth sport.

## 1 Introduction

Poor wellbeing among children and adolescents is an increasing concern [1]. Research reviews and longitudinal studies generally indicate that participation in leisure‐time club‐based sports has a positive impact on young participants’ wellbeing [2, 3, 4, 5]. However, studies also reveal that this is not always the case and that some aspects of sports environments can have detrimental effects on wellbeing among young participants [6, 7, 8].

In Denmark and other Scandinavian countries, leisure‐time youth sport is mainly organized by volunteer coaches in local sports clubs affiliated with regional and national organizations. As Denmark's largest organization for children and adolescents with more than 870 000 members under the age of 18, the National Olympic Committee and Sports Confederation of Denmark (DIF) has a responsibility to ensure that club sport environments promote wellbeing among children and adolescents. To support this effort, DIF hosted a consensus conference with the aim of gathering knowledge and establishing a shared understanding of what characterizes wellbeing in youth sport and how it can be promoted.

## 2 Methodology and Procedures

A 2‐day consensus conference was held in Copenhagen, Denmark, on August 29 and 30, 2024. The consensus panel was selected and invited by DIF. It consisted of seven researchers from various disciplines and six expert practitioners from Denmark, all of whom have extensive knowledge about and experience with promoting wellbeing in and through youth sport. The following disciplines were represented: Sports psychology, public health promotion, anthropology, sociology, and exercise physiology. The expert practitioners were selected to include practitioners with extensive practical experience from working with wellbeing promotion among youth in various settings such as local sport clubs, sport federations, educational settings, psychiatry, sport psychological consulting, and NGOs. On demographics, the panelists had an even gender distribution, and the age ranged from mid‐thirties to mid‐sixties.

The consensus process aimed to integrate international research‐based knowledge with practice‐based knowledge into key factors influencing wellbeing in youth sport. A special focus was devoted to how wellbeing in and through sport can be promoted within the context of Danish leisure‐time club sport. While this limitation is noticeable, several statements may also be relevant beyond the specific geographical and cultural setting.

The consensus process was led by one of the coauthors who has led previous consensus conferences [2]. The consensus process included the following steps. At first, each of the 13 panelists presented their knowledge, experiences, and perspectives on wellbeing in youth sport. Secondly, panelists identified the main topics that they were to form consensus statements on, resulting in the four consensus questions described below. For each of these main consensus questions, the more specific substatements were formed in a two‐step process: Panelists were divided into three subgroups that each had different research and practical backgrounds represented. In these groups, participants’ knowledge and experience about the question addressed were presented and discussed, and the subgroups’ suggestions for statements were then formed. These were then presented and argued for to the other groups. The total pool of the groups’ statements was then discussed and negotiated in plenum, leading to those that were similar or overlapping being merged, while those that were disputed by others were discussed and negotiated in depth. The guiding principle for final inclusion or exclusion of disputed statements was that all participants did not have to find it ideal or be in 100% agreement, but all panelists had to find the statement acceptable for a specific statement to be included. Often, disputed statements were accepted through slight changes of wording, but some statements important to some, but unacceptable to others, were also excluded.

The consensus process and the resulting statements were based on the premise that children and young people's wellbeing depends on structural conditions and resources and is developed in interaction with their environment. This system approach to wellbeing in youth sport and how it can be promoted was developed through formulating and discussing statements related to wellbeing for children and adolescents, while considering also the significance of their sports environments, community factors, and policy (recommendations) in line with the socioecological model [9, 10]. The following four questions were identified:

1. What characterizes wellbeing in sport for children and adolescents?
2. What characterizes sport environments that promote children and adolescents’ wellbeing?
3. Which factors are important to consider in efforts to develop wellbeing‐promoting youth sport environments?
4. How can wellbeing in youth sport be promoted?

All consensus conference participants were involved in answering and agreeing on an answer to the questions, drawing on their own research, prior readings, experiences from practice, and a (yet unpublished) literature review that was made by external consultants to support this specific purpose. While answers to the first two questions are based on the literature, the last two questions were answered through a more analytical process. This involved first identifying aspects of the surrounding sociocultural community that affect local youth sport environments and then describing important concrete actions based on all the agreed statements.

## 3 Statements

### 3.1 Question 1: What Characterizes Wellbeing in Sport for Children and Adolescents?

In the research literature, many differing conceptualizations, definitions, and synonyms of wellbeing exist, ranging from feelings of positive affect or perceived quality of life to the absence of mental health challenges. One important distinction is between wellbeing as feeling well, often described as subjective (or hedonic) wellbeing, and wellbeing as functioning well, often described as psychosocial (or eudemonic) wellbeing [11]. Another important distinction is between context‐specific wellbeing, such as wellbeing in the sport context itself, and general wellbeing in life [11], which may be affected through sports participation. These differences reflect that wellbeing is a very broad concept consisting of various aspects. One way to combine these different aspects in one sentence is to define wellbeing as a state of being, feeling, and functioning well [12, 13, 14]. However, it is important to maintain and consider that interdependent dimensions are involved in wellbeing in and through activities such as youth sport. Social, mental, and physical/bodily wellbeing are three often used aspects [15], which are relevant to consider when examining the impact of sport participation, as sport constitutes physical activities embedded within social contexts that elicit and modulate cognitive and emotional processes.

Connecting these three aspects of sport implies that wellbeing in sport occurs when the participants can actively engage, express, and develop themselves in and through sports, while experiencing bodily, social, and mental wellbeing.

*Social wellbeing* in sport is characterized by children and adolescents feeling included in safe and committed communities and developing positive relationships [16, 17].

*Mental wellbeing* in youth sport is characterized by children and adolescents experiencing autonomy, competence, joy of movement, and togetherness [18, 19].

*Bodily wellbeing* in youth sport is characterized by children and adolescents experiencing the capacities and potentials of their bodies, developing new skills, and physical wellbeing [4, 20].

### 3.2 Question 2: What Characterizes Sport Environments That Promote Children and Adolescents’ Wellbeing?

- Children and adolescents’ wellbeing in sport is higher in sport environments that have task involvement goals, effort, learning, and development as success criteria [6, 7, 21].
- Children and adolescents experience greater wellbeing in sports environments that minimize performance pressure and emphasize personal growth over ego‐involving competition [6, 7, 8].
- Children and adolescents' wellbeing in sport is higher in sport environments in which everyone has access to contribute and feel valued in the community [7, 16].
- Children and adolescents' wellbeing in sport is higher in sport environments that are safe spaces where children and adolescents can be themselves, have their voices heard, and make choices while also sharing the responsibility [19, 22].
- Children and adolescents' wellbeing in sport is higher in child‐centric sport environments that respect children's rights and developmental needs through organizing age‐appropriate training and competition regarding both amount/volume and content/activities [23].

The above may be achieved by having responsible and nurturing coaches and leaders who foster cooperation, cohesion, and have a learning‐oriented and experimental/playful approach to training [7]. Furthermore, sports environments may promote wellbeing of children and adolescents through defining common values for how to behave toward each other, and by promoting inclusivity and diversity through varied participation opportunities [24].

### 3.3 Question 3: Which Factors Are Important to Consider in Efforts to Develop Wellbeing Promoting Youth Sport Environments?

To develop sport environments that promote wellbeing, it is important to consider how the above‐mentioned characteristics are shaped by and hence dependent on a number of factors associated with the wider sociocultural community in which the sport club is situated. These are interdependent factors at the individual, interpersonal, community, and macro/policy level.

- Important factors to consider at the individual level are the young athletes' age, interests, skills, maturity, and life conditions and challenges, along with coaches and managers' skills, experiences, and values, and not least parents' interests, involvement, support, and values.
- Important factors at the interpersonal level are the social relations between participants – inside and outside of the sporting activity; relationships between the young athletes and their coaches and leaders; relationships between coaches and the wider team of coaches; and relationships between the sports community and the parents/private homes.
- Important factors at the community level are the values, culture, and cohesion of the sports community; the connection between the sports community and other arenas and institutions in the local community, such as schools, afterschool daycare, and businesses; access to local sports facilities; and socioeconomic support for children and young people to participate in sport.
- Important factors at the macro/policy level are allocation of finances and other resources for child and youth sports; action plans for how sports communities can sustainably support wellbeing; political governance that ensures children and young people's rights to protection and age‐appropriate provision of sport and participation options therein.

### 3.4 Question 4: How Can Wellbeing in Youth Sport Be Promoted?

Stakeholders at all levels can contribute to promoting/ensuring wellbeing in youth sport. The following statements are the end results of the consensus process discussing what wellbeing is in youth sport (described in Theme 1), current knowledge about the characteristics of sport environments that promote wellbeing in youth sport (described in Theme 2), and factors at the different socioecological levels that shape such characteristics (described in Theme 3).

Young sport participants can promote wellbeing in youth sport by:

- Being inclusive, respectful, and showing support and care toward all participants on the team, especially newcomers or those who seem to struggle or feel insecure.
- Showing respect and regard for opponents/competitors, referees, and coaches.
- Not criticizing or showing dislike of others' performances or mistakes.

Parents/caretakers can promote wellbeing in youth sport by:

- Being respectful and supportive toward all participants, opponents, referees, and coaches.
- Acknowledging effort, development, and mastery as the main success criteria.
- Asking their child about experiences and learnings before results.
- Taking responsibility and contributing to the sports communities.
- Aligning their expectations with the aims, values, and possibilities provided by the sport clubs.

Coaches and leaders/managers can promote wellbeing in youth sport by:

- Developing sports environments with a task‐involving atmosphere by focusing on effort, development, and mastery.
- Encouraging teamwork with shared values and approaches.
- Supporting collaboration among children and adolescents both inside and outside of sporting activities.
- Establishing inclusive training environments that bring together children and adolescents across different backgrounds and skill levels.
- Ensuring that training is varied, age‐appropriate, and differentiated, making sports meaningful and stimulating for all participants.

Local sports communities can promote wellbeing in youth sport by:

- Ensuring and helping coaches and managers develop relevant and sufficient relational, sporting, pedagogical, and managerial skills and experience.
- Ensuring the rights of children and adolescents and developing action plans to ensure sustainable development of their wellbeing.
- Develop a policy on wellbeing in youth sports and consider implementing regulations that safeguard the wellbeing of children and adolescents.

At a macro policy level, sports federations can promote wellbeing in youth sport by:

- Creating tournament structures and sports programs that accommodate diverse ways of engaging in sport.
- Systematically work to create and integrate knowledge about the wellbeing of children and young people into training, dissemination, and educational activities.
- Develop a unified policy on wellbeing in youth sports and consider introducing regulations to safeguard the wellbeing of children and adolescents.

## 4 Limitations

A main limitation of these consensus statements is that they are primarily developed as a knowledge foundation for promoting wellbeing in the context of Danish youth sport. This has shaped which research and practice‐based knowledge was found relevant to include and the focus in the practical implications on the sports club setting. Another limitation is that the research that has been selected and cited as the primary knowledge sources is mainly quantitative studies or literature reviews that synthesize quantitative studies. Thus, the current consensus statements may be supplemented by broader studies and qualitative research where children describe their experiences of wellbeing in sport with their own words and perspectives.

## Conflicts of Interest

The authors declare no conflicts of interest.

## Data Availability

Data sharing not applicable to this article as no datasets were generated or analysed during the current study.
